# A novel diffuse large B-cell lymphoma-associated cancer testis antigen encoding a PAS domain protein

**DOI:** 10.1038/sj.bjc.6601875

**Published:** 2004-05-25

**Authors:** A P Liggins, P J Brown, K Asker, K Pulford, A H Banham

**Affiliations:** 1 Nuffield Department of Clinical Laboratory Sciences, University of Oxford, LRF Immunodiagnostics Unit, Level 4 Academic Block, John Radcliffe Hospital, Oxford, Oxfordshire OX3 9DU, UK

**Keywords:** cancer testis antigen, lymphoma, SEREX, PAS domain

## Abstract

Here we report that the OX-TES-1 SEREX antigen, which showed immunological reactivity with serum from four out of 10 diffuse large B-cell lymphoma (DLBCL) patients, is encoded by a novel gene, *PAS domain containing 1* (*PASD1)*. *PASD1_v1* cDNA encodes a 639 amino-acid (aa) protein product, while an alternatively spliced variant (*PASD1_v2*), lacking intron 14, encodes a 773 aa protein, the first 638 aa of which are common to both proteins. The PASD1-predicted protein contains a PAS domain that, together with a putative leucine zipper and nuclear localisation signal, suggests it encodes a transcription factor. The expression of *PASD1_v1* mRNA was confirmed by RT–PCR in seven DLBCL-derived cell lines, while *PASD1_v2* mRNA appears to be preferentially expressed in cell lines derived from non-germinal centre DLBCL. Immunophenotyping studies of *de novo* DLBCL patients' tumours with antibodies to CD10, BCL-6 and MUM1 indicated that two patients mounting an immune response to PASD1 were of a poor prognosis non-germinal centre subtype. Expression of *PASD1* mRNA was restricted to normal testis, while frequent expression was observed in solid tumours (25 out of 68), thus fulfilling the criteria for a novel cancer testis antigen. PASD1 has potential for lymphoma vaccine development that may also be widely applicable to other tumour types.

Diffuse large B-cell lymphoma (DLBCL) is the most common form of adult non-Hodgkin's lymphoma and is heterogeneous with respect to morphology, clinical features and immunophenotype (Jaffe *et al*, 2001). Approximately 50% of DLBCL patients relapse after conventional anthracycline-based CHOP-type treatment (The Non-Hodgkin's Lymphoma Classification Project, 1997). Gene expression studies have identified clinically-relevant subtypes of DLBCL, showing that patients with a germinal centre-derived tumour have an improved prognosis compared to those with a non-germinal centre-derived tumour (Alizadeh *et al*, 2000; Rosenwald *et al*, 2002). Improving the outcome for these patients requires both the identification of high-risk patients at diagnosis and the development of alternative effective therapeutic strategies.

There is increasing evidence for the existence of an anti-tumour immune response against proteins expressed by malignant cells. Antibodies to tumour-associated proteins have been found in the blood of cancer patients and these antibodies can be exploited to enable the identification of tumour-associated proteins using SEREX (serological analysis of recombinant cDNA expression libraries). This technique has been used in a number of laboratories to identify more than 2000 tumour-associated antigens (Türeci *et al*, 1999; Preuss *et al*, 2002). Since these antigens have included molecules that were originally identified by cloning cytotoxic T lymphocyte (CTL)-recognised epitopes, for example, MAGE-1 and tyrosinase (van der Bruggen *et al*, 1991; Brichard *et al*, 1993; Sahin *et al*, 1995), SEREX can be used to detect tumour antigens eliciting both cellular and humoral immunity.

Cancer testis antigens (CTAs) constitute some of the most promising tumour-associated antigen candidates for therapeutic development, being normally expressed only in immunologically-privileged sites such as the testis, but also in neoplastic cells (Scanlan *et al*, 2002). Examples of CTAs currently being used for immunotherapy include NY-ESO-1 and MAGE-A3 peptides in melanoma (Coulie *et al*, 2001; Jäger *et al*, 2001). However, it should be noted that other categories of SEREX antigens, such as the overexpressed HER-2/neu protein, are being targeted with some success in the treatment of breast carcinomas (Bernhard *et al*, 2002). SEREX antigens can also be used as adjuvants to boost the immune response to other tumour antigens (Nishikawa *et al*, 2001).

We have previously used SEREX to identify 28 proteins whose expression might be relevant to the pathogenesis of DLBCL, and which may provide potential novel immunotherapeutic targets (Liggins *et al*, 2004). One of the antigens, OX-TES-1, was recognised by serum from four out of 10 patients with DLBCL, one out of 20 normal serum samples and no acute myeloid leukaemia (AML) or chronic myeloid leukaemia (CML) serum samples (*n*=10 of each). This report describes the sequence and genomic organisation of the gene encoding this antigen*, PAS domain containing 1* (*PASD1*), and the identification of an alternatively spliced variant, *PASD1_v2*, both variants being expressed in DLBCL cell lines. Expression studies indicate that *PASD1* is a novel CTA showing widespread mRNA expression in patients with solid tumours and in DLBCL-derived cell lines.

## MATERIALS AND METHODS

### Tissue samples

This project was approved by the Oxford Clinical Research Ethics Committee and informed patient consent was obtained prior to the collection of serum samples used in the SEREX screening. Tissue biopsies from the DLBCL patients were obtained from the Department of Pathology, John Radcliffe Hospital, Oxford.

### Cell lines and culture conditions

The OCI-Ly3, OCI-Ly10 (activated B-cell-derived), SUDHL-6, SUDHL-10 and DB (germinal centre-derived) DLBCL cell lines were a kind gift from Dr Eric Davis and Dr Andreas Rosenwald, Bethesda, MD, USA, and the LIB, MIEU, DEAU and HLY-1 DLBCL cell lines were generously provided by Dr Talal Al Saati, Toulouse, France. These cell lines were maintained in RPMI 1640 medium (Sigma Aldrich, UK) supplemented with 10% foetal calf serum and antibiotics (penicillin (5000 U ml^−1^) and streptomycin (5000 *μ*g ml^−1^), Invitrogen, UK) in an atmosphere of 5% CO_2_ at 37°C. Cells were washed in RNase-free PBS prior to mRNA extraction.

### OX-TES-1 expression cloning and sequencing

A single cDNA clone encoding the full-length OX-TES-1 antigen was isolated as previously described using SEREX (Liggins *et al*, 2004). The cDNA insert was commercially sequenced to publication quality by MWG Biotech (GenBank Accession Number AY270020).

### Hybridisation of the *PASD1* cDNA to expression arrays

Multiple tissue expression (MTE) and matched tumour/normal (MTN) arrays (BD Biosciences Clontech, CA, USA) were prehybridised according to the manufacturer's instructions. A 610 bp *Pst*1 fragment of *PASD1_v1*, also present in the splice variant (*PASD1_v2*) and representing a portion of the 3′ UTR, was radiolabelled using the High Prime DNA Labelling Kit (Roche Molecular Biochemicals, UK). This probe was hybridised to both arrays, according to the manufacturer's instructions, before exposure to film at −70°C for 27 days. Additional information about the tissues and cases on these arrays can be obtained from the BD Biosciences Clontech website (www.bdbiosciences.com/clontech). Loading of the cDNAs on the MTN and MTE arrays is normalised for three and eight housekeeping genes respectively to enable quantitative comparisons between gene expression in different tissues. As an additional loading control, a radiolabelled ubiquitin cDNA probe was subsequently hybridised to the stripped array according to the manufacturer's instructions.

### Reverse transcription–polymerase chain reaction

Reverse transcription–polymerase chain reaction (RT–PCR) was carried out as follows: Poly(A)+mRNA from the seven DLBCL-derived cell lines was extracted using *μ*MACS mRNA Isolation kits (Miltenyi Biotech, Germany). The cDNA was reverse transcribed at 42°C for 50 min from 20 ng mRNA in a 25 *μ*l reaction containing 200 U Superscript II™ RNase H^−^ reverse transcriptase (Invitrogen, UK), 1 × First Strand Buffer, 4 mM DTT and 100 ng of either oligo(dT) primer or random hexamers. The integrity of cDNA templates was assessed using gene-specific primers to *β*-actin. 2 *μ*l of cDNA was amplified in a 25 *μ*l PCR reaction containing 200 *μ*M each dNTP, 4 *μ*M each primer, 1 × PCR buffer and 1 × Advantage 2 Polymerase mix (BD Biosciences Clontech, CA, USA). Gene-specific primers were designed to amplify fragments of 211–1505 bp. Primer pair A: forward: 5′-TACAGGAGCGGAAGAAGTGG-3′; reverse: 5′-ACAGGAACAATGGGTTGGG-3′; primer pair B: forward: 5′-TCTCATCAATAGCAACTTGCTC-3′; reverse: 5′- TCACACTCACTTCCCTCTTAC-3′; and primer pair C: forward: 5′-TCCAGAGAGCAGGCTGAACAA-3′, reverse: 5′-AAGCCGGATGTAATCCTGTG-3′. Cycling parameters were as follows: 5 min at 94°C (initial denaturation) then 45 s at 94°C, 45 s at appropriate annealing temperature (A 60°C, B 55°C, C 60°C) and 2.5 min (A and B) or 5 min (C) at 72°C for 30 (A and B) or 35 (C) cycles. Phagemid DNA containing the appropriate cDNA insert was used as a positive control, while the negative control was a PCR mixture with no cDNA template. Reactions were also carried out on cDNA synthesis reactions that lacked reverse transcriptase. PCR products were visualised after separation in agarose gels by staining with ethidium bromide.

### Immunohistochemistry

Formalin-fixed paraffin-embedded sections from the DLBCL biopsies were dewaxed/rehydrated and then antigen retrieval was performed by microwave pressure cooking for 3 min in 50 mM Tris; 1 mM EDTA (pH 9). Immunostaining was performed using the DAKO Envision system with primary antibodies to CD10 (Novocastra), BCL-6 (DAKO) or MUM1 (a kind gift from Professor B Falini, Perugia). The stained sections were counterstained with haematoxylin and mounted in Aquamount (VWR).

## RESULTS

The single cDNA clone encoding the OX-TES-1 antigen was identified by screening a testis library with serum from a single patient with aggressive DLBCL as previously described (Liggins *et al*, 2004). The 4.2 kb cDNA insert from the phagemid was fully sequenced. Sequence searches at the time the gene was cloned, using the BLAST search engine at the National Center for Biotechnology Information, indicated that the cDNA sequence represented a novel gene that had not been deposited in the public databases.

### Sequence analysis of *PASD1* and identification of an alternatively spliced form, *PASD1_v2*

**Figure 1:**
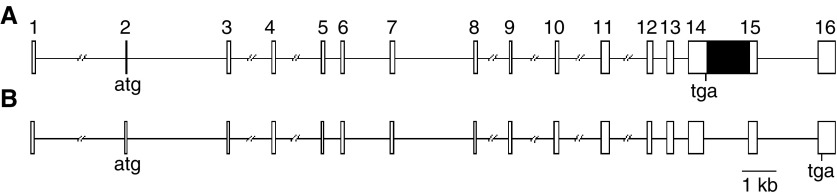
Genomic structure of *PASD1_v1* (**A**) and *PASD1_v2* (**B**). Exons are indicated as open boxes, introns as lines and the retained intron in *PASD1_v1* is indicated with a black box. The position of predicted translational start and stop sites are indicated.

A UniGene folder (Hs.160594) has recently been created for this gene, which encodes an unnamed protein product and maps to Xq28. The HUGO gene nomenclature committee has named the gene *PAS domain containing 1* (*PASD1*). A recently-sequenced human testis MGC clone (BC040301) contains a smaller cDNA (2850 bp) encoding a longer PASD1 protein product that we have named PASD1_v2. Subsequently, when specifically referring to our variant, we use the name PASD1_v1. A comparison of both the *PASD1_v1* and *PASD1_v2* cDNA sequences with the human genome sequence demonstrated that these are alternatively spliced, with the 1.27 kb sequence corresponding to intron 14 being retained within the *PASD1_v1* transcript (Figure 1

).

**Figure 2:**
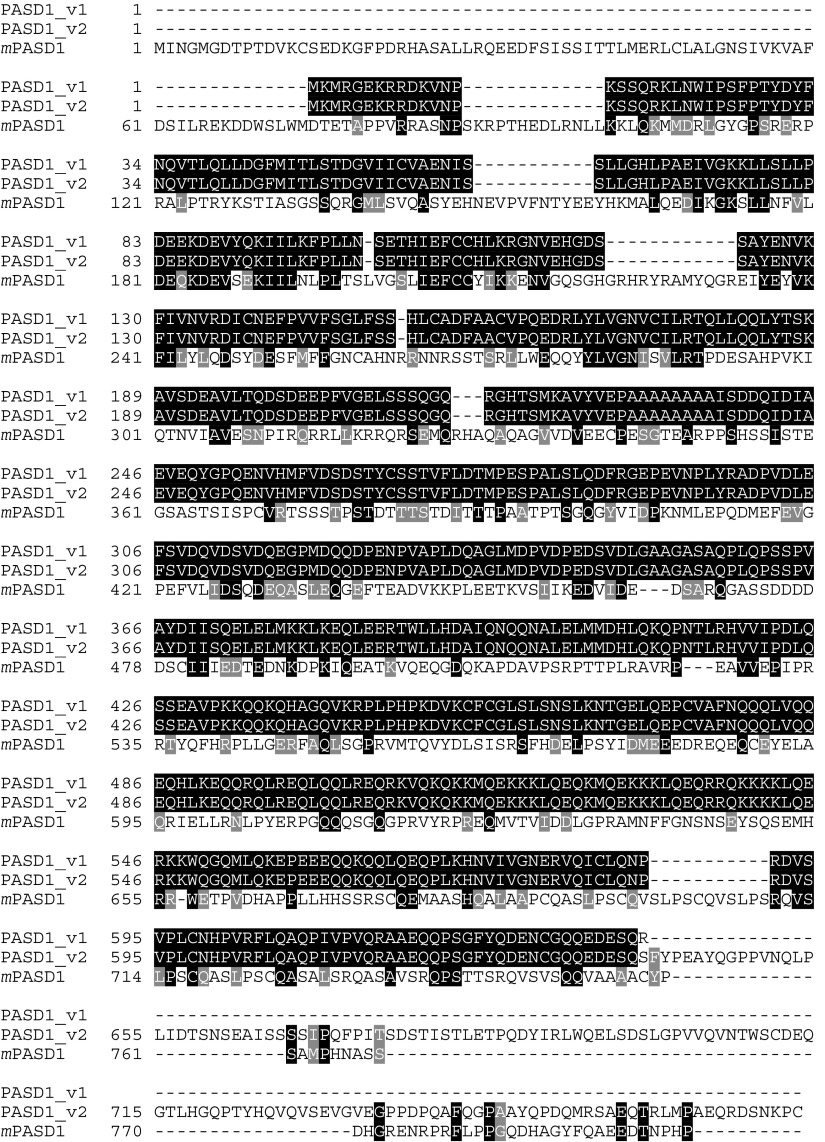
PASD1_v1 and PASD1_v2 proteins, along with the murine homologue, *m*PASD1. Identical residues are highlighted while similar residues are shaded in grey. The murine protein shows 35.7% similarity (25.2% identity) with PASD1_v1 and 34.1% similarity (24.2% identity) with PASD1_v2.

Translation of the *PASD1* cDNA sequence predicted the existence of two potential methionine start codons for this protein; since the second (aa3) has a slightly better Kozak consensus sequence (Kozak, 1987) than the first, it may represent the start of translation. Both *PASD1_v1* and *PASD1_v2* encode a predicted protein product with identical N-terminal sequence (638 aa). The retained intron in *PASD1_v1* introduces a stop codon after aa 639, while *PASD1_v2* encodes an additional 134 aa at the C-terminus (Figure 2

). The poly(A) tail and the presence of upstream stop codons before the translated protein sequence indicated that a full-length *PASD1_v1* cDNA had been isolated. A hypothetical gene similar to LOC139135 in UniGene Mm.295937 may represent the murine homologue of PASD1 (Figure 2); this gene also maps to the X chromosome.

### Sequence analyses of the PASD1 protein

**Figure 3:**
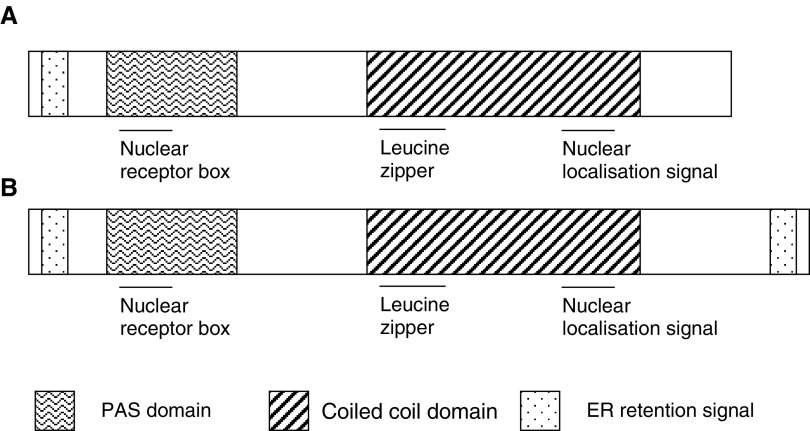
Schematic illustration of the domains within the PASD1_v1 (**A**) and PASD1_v2 (**B**) proteins (not to scale). Additional domains are underlined.

Analyses of the human PASD1 protein sequence using databases on the World Wide Web predict the presence of a number of domains, several of which are illustrated in Figure 3

. Analysis using the programme MotifFinder identified two overlapping N-terminal PAS (Per ARNT Sim) domains (using the Pfam and Prosite databases) between aa 32–94 and aa 41–137. Analysis using the PSORT II programme identified an R-2 motif at aa 14 (which is a predicted cleavage site for mitochondrial presequence), a nuclear localisation signal at aa 539, an ER membrane retention signal at the N-terminus, a C-terminal leucine zipper pattern between aa 482–503 and a coiled-coil domain between aa 476–557. An LXXLL motif or nuclear receptor (NR) box was also detected in the N-terminus of the PASD1 protein between aa 77–81. In addition to the domains illustrated, there is a proline-rich region between aa 478–639, a lysine-rich region between aa 508–548, and a glutamine-rich region between aa 479–638. There is only one domain, an ER membrane retention signal between aa 769–772, in the additional region encoded by PASD1_v2.

Database searches using the PASD1 protein sequence have shown that the most closely related proteins, other than the murine homologue, are the neuronal PAS domain protein 2 of the zebrafish *Danio rerio* (34% identity) and the CLOCK protein of the Korean rock fish *Sebastes schlegeli* (32%).

### Expression of *PASD1* mRNAs in normal human tissues

**Figure 4:**
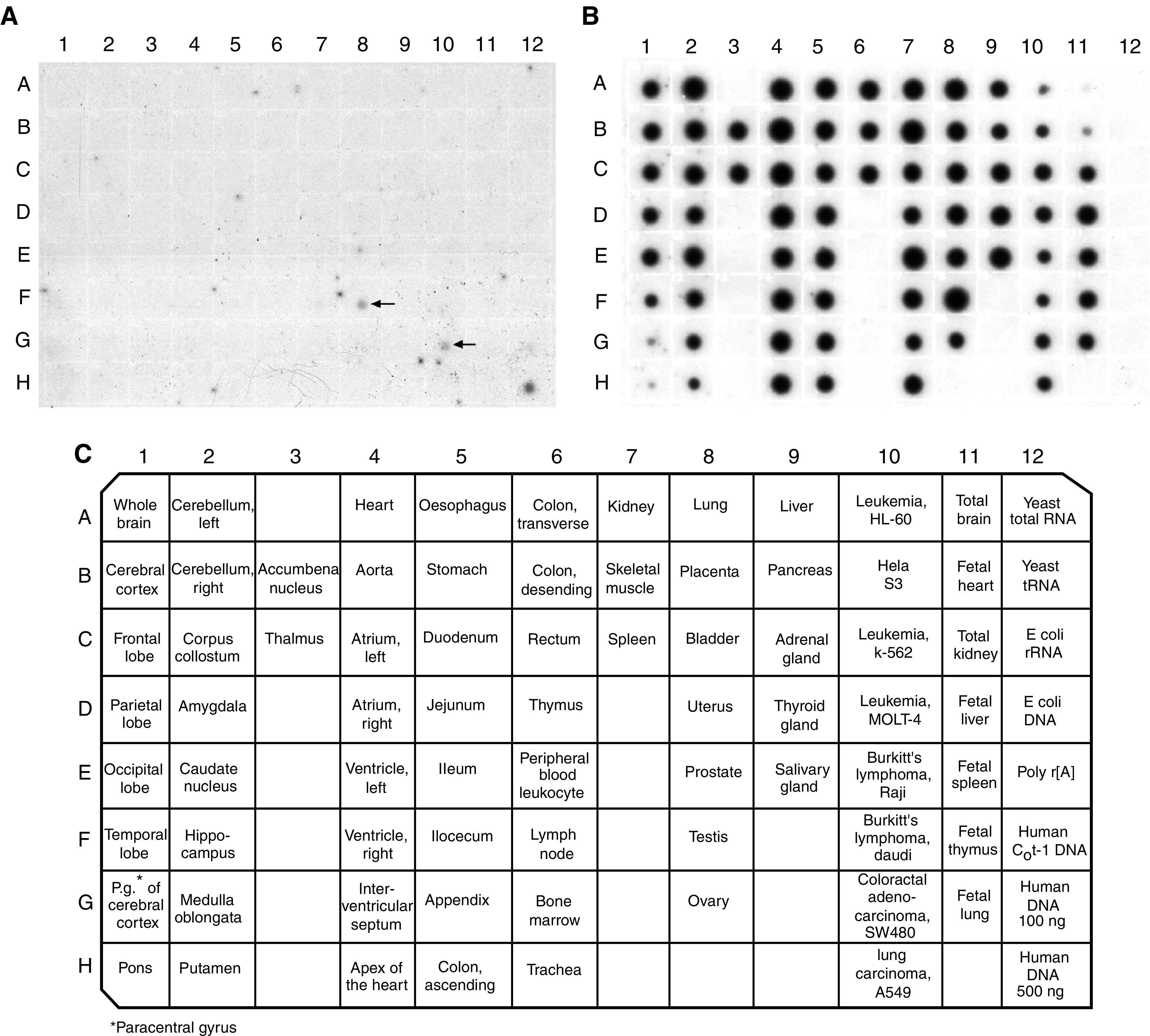
Results obtained from hybridising the *PASD1* cDNA (**A**) or the ubiquitin cDNA control probe (**B**) to BD Biosciences' MTE array. The identity and position of tissues on the array is shown in the lower panel (**C**). The positive signals for *PASD1* are arrowed.

A normal tissue-derived MTE cDNA array, prepared from pooled individuals that are non-diseased victims of sudden death/trauma, was probed with a cDNA fragment that is common to both *PASD1_v1* and *PASD1_v2*. The expression of the *PASD1* mRNA in both adult and foetal normal human tissues was shown to be restricted to testis (Figure 4A

). This gene is expressed at very low levels requiring a long exposure time to obtain the data illustrated and repetition confirmed this result. Our expression data are supported by the normal tissue sources of EST sequences in UniGene folders Hs.160594 and Mm.295937, all of which have been isolated from testis (or pooled samples containing testis). Furthermore, both the *PASD_v1* and *PASD1_v2* cDNAs were isolated from testis cDNA libraries. The other positive signals seen on this array are those from a colorectal adenocarcinoma cell line and the human DNA (500 ng) control.

### Expression of *PASD1* mRNAs in neoplastic tissues

**Figure 5:**
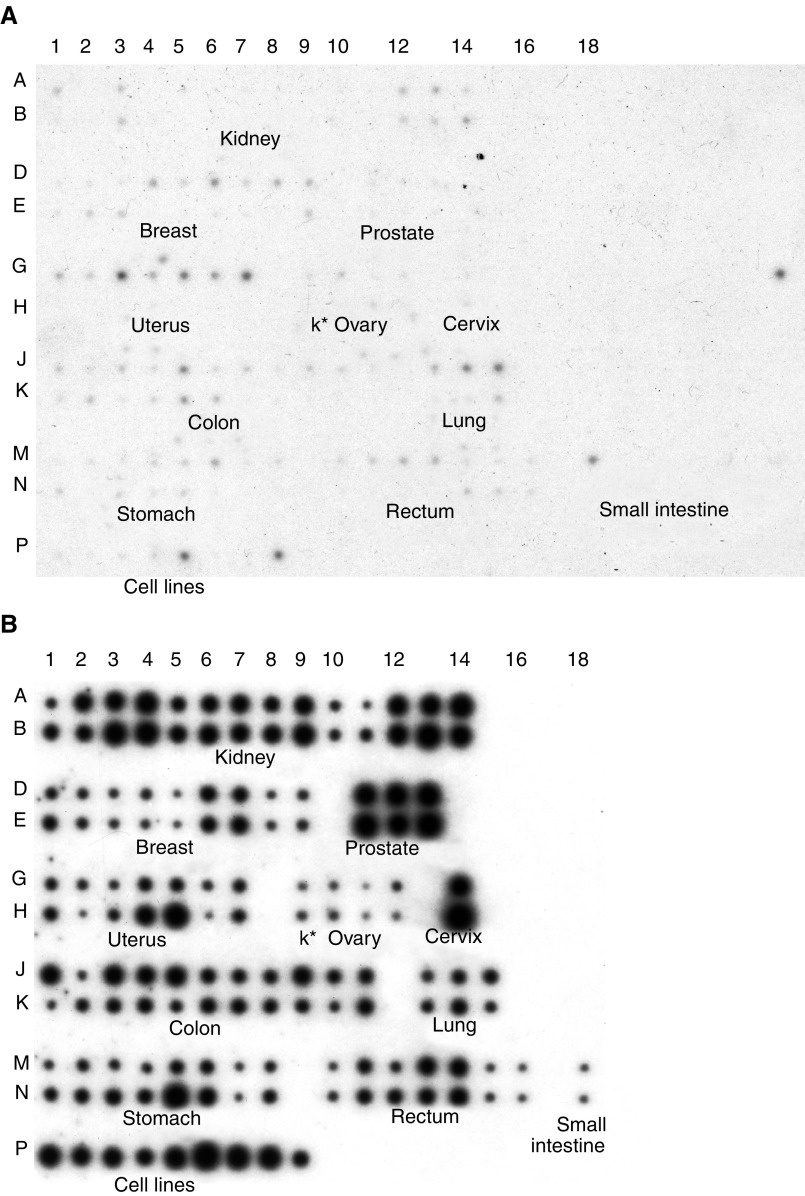
Results obtained from hybridising the *PASD1* cDNA (**A**) or the ubiquitin cDNA control probe (**B**) to BD Biosciences' MTN expression array. The identity and position of tissues on the array are shown. Rows A, D, G, J, and M are normal tissue while B, E, H, K, an N are tumour tissue, K^*^=kidney. Row P indicates the human cancer cell lines: 1 – HeLa, 2 – Daudi (Burkitt's lymphoma), 3 – K562 (chronic myeloid leukaemia), 4 – HL-60 (promyelocytic leukaemia), 5 – G361 (melanoma), 6 – A549 (lung carcinoma), 7 – MOLT-4 (lymphoblastic leukaemia), 8 – SW480 (colorectal adenocarcinoma), and 9 – Raji (Burkitt's lymphoma).

The same cDNA probe was used to analyse the expression of *PASD1* mRNA in human tumours and adjacent histologically-normal tissue (taken from the same cancer patients) on the MTN array (Figure 5A

). Although *PASD1* expression was restricted to testis in normal individuals, the histologically ‘normal’ tissues were frequently found to express this gene. In some tissues, most notably breast, uterus, small intestine and lung, there was often higher expression in the ‘normal’ tissues than was observed in the paired tumour tissue. The results obtained after rehybridising the array with a ubiquitin probe confirmed that this was not caused by differences in sample loading (Figure 5B). A possible explanation for this observation is that changes in PASD1 expression may be an early event in carcinogenesis that occurs before histological changes are apparent. The *PASD1* mRNA is shown to be expressed in 25 of the 68 solid tumour tissues included on the MTN array. *PASD1* expression was also seen in the human cancer cell lines (Figure 5A, Row P), with the melanoma (P5) and colorectal adenocarcinoma (P8) cell lines showing the highest expression levels. Incidentally, the colorectal adenocarcinoma cell line on the MTN array is the same as that on the MTE array, where it also gave a positive signal (Figure 4A). Other cell lines common to both arrays showed very weak expression on the MTN array, while no signal was observed on the normal tissue MTE array. However, probing the arrays with ubiquitin as a loading control indicated that the cDNAs for these cell lines were under-represented on the MTE array.

### Expression of the *PASD1_v1* and *PASD1_v2* mRNAs in DLBCL cell lines

**Figure 6:**
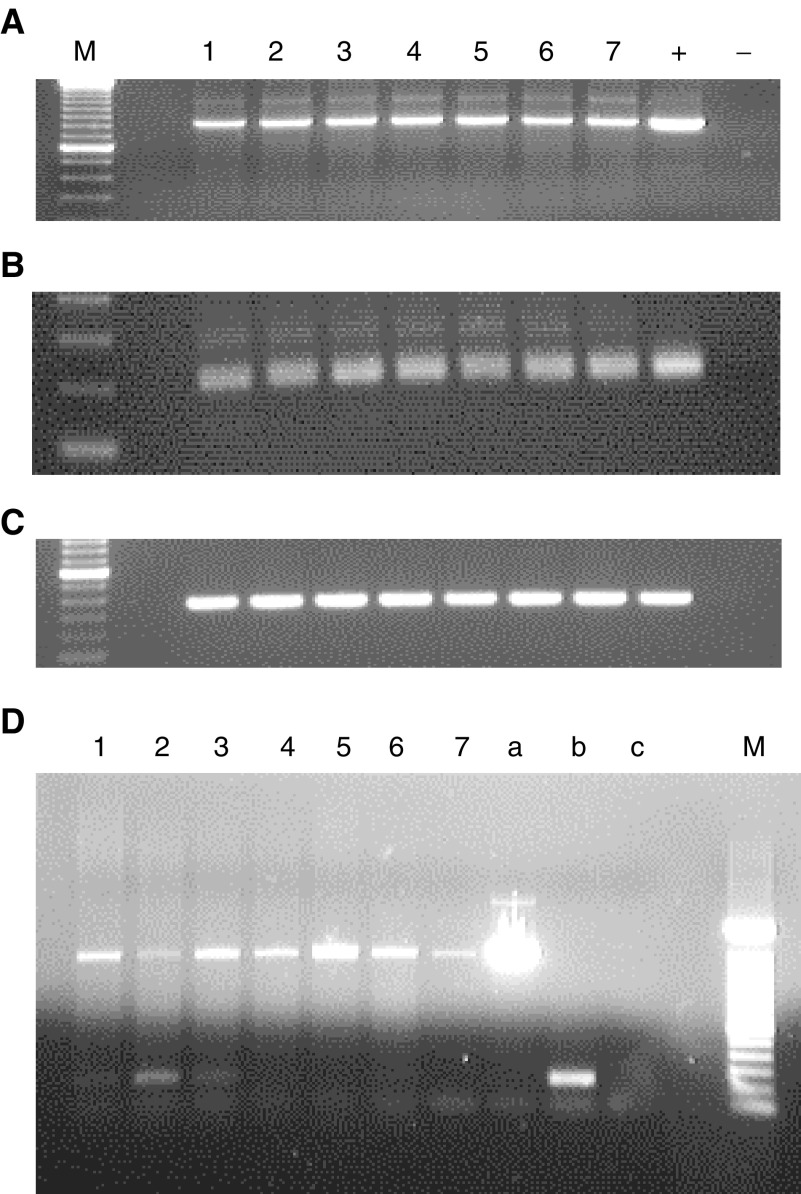
*PASD1* products amplified by RT–PCR from DLBCL cell lines: (**A**): *β*-actin control; (**B**): PCR with primer set A (detects both transcripts, same size product); (**C**): PCR with primer set B (*PASD1_v1* specific): M=100 bp ladder; 1–7=cell lines (in order): OCI-Ly3; OCI-Ly10; HLY-1; SUDLH6; MIEU; LIB; DEAU; +=positive control; −=negative control; (**D**): PCR with primer set C (*PASD1_v1* large product and *PASD1_v2* small product) M=100 bp ladder; 1–6=cell lines (in order): OCI-Ly3; OCI-Ly10; HLY-1; SUDLH6; SUDHL10; DB; 7=genomic DNA; a=*PASD1_v1* positive control; b=*PASD1_v2* positive control; *c*=negative control.

Since the *PASD1* cDNA was originally cloned from a testis cDNA library, RT–PCR was used to confirm whether the *PASD1* mRNA was expressed in a panel of DLBCL-derived cell lines. Products were obtained with all cell lines using primers to *β*-actin (Figure 6A

), indicating the integrity and suitability of the cDNAs as templates for PCR. Analysis of *PASD1* mRNA expression with primer set A successfully amplified fragments of the same size as the positive control (211 bp) in all DLBCL cell lines tested (Figure 6B). Primer set A was designed to amplify a region of cDNA common to both *PASD1_v1* and *PASD1_v2* and therefore does not distinguish these two mRNA species.

To confirm the expression of the *PASD1_v1* mRNA, RT–PCR was carried out with primers designed to the retained intronic sequence that is absent in *PASD1_v2* (primer set B). Figure 6C shows that a fragment of the same size as the positive control (360 bp) was successfully amplified in all DLBCL cell lines. This confirms that the *PASD1_v1* mRNA is transcribed in DLBCL-derived cell lines.

The *PASD1_v2* cDNA does not contain any unique sequence that could be used to determine whether both variants are transcribed in DLBCL. Therefore, RT–PCR was carried out with primers designed to regions in exons 14 and 15 that flank the retained intronic sequence (primer set C). With these primers, a 1505 bp fragment indicates the expression of *PASD1_v1*, while a 238 bp fragment indicates the expression of *PASD1_v2*. Figure 6D shows that a product of ∼1500 bp is amplified in all the DLBCL cell lines. A smaller product of ∼240 bp, was observed in the OCI-Ly3, OCI-Ly10 and HLY-1 cell lines suggesting that *PASD1_v2* is transcribed in DLBCL-derived cell lines of a non-germinal centre phenotype. No products were obtained when PCR was performed on cDNA synthesis reactions that lacked reverse transcriptase (data not shown) indicating that there was no genomic DNA contamination and the products obtained are amplified from cDNA.

### Immunophenotyping of patients

In the original SEREX study, serum from 10 DLBCL patients was tested for the presence of antibodies to the PASD1 protein. Three of these patients had DLBCL transformation from follicular lymphoma, which generally has a poor prognosis; one of these patients showed a humoral immune response to the PASD1 protein. The remaining patients, including the patient whose serum sample was used for SEREX screening, were diagnosed with *de novo* DLBCL. According to Hans *et al* (2004), immunohistochemical labelling using monoclonal antibodies to the germinal centre markers CD10, BCL-6 and the non-germinal centre marker MUM1 can be used to distinguish good prognosis (germinal centre) and high-risk (non-germinal centre) derived subtypes of *de novo* DLBCL (Table 1

**Table 1:** Scoring system for subtyping *de novo* cases of DLBCL

CD10	+	−	−	−
BCL-6		−	+	+
MUM1			−	+
Subtype	GC	non-GC	GC	non-GC

Cases were scored as positive when more than 30% of the tumour cells were positive. GC=germinal centre and non-GC=non-germinal centre. Empty boxes reflect that the presence or absence of this marker does not influence the scoring outcome.

). Paraffin-embedded DLBCL biopsy sections were available for subtyping six of the seven patients with *de novo* DLBCL. The tumour from the patient whose serum was used for library screening was negative for CD10 and BCL-6 and positive for MUM1, suggesting that the patient had a poor prognosis non-germinal centre-derived subtype of DLBCL; this is consistent with the short survival time. Of the remaining five *de novo* DLBCL patients, four appeared to be non-germinal centre-derived, while the other was germinal centre-derived. Thus, the two *de novo* patients that showed serum reactivity with PASD1 were of a non-germinal centre-derived subtype of DLBCL. No biopsy material was available for the fourth patient mounting a humoral immune response to the PASD1 protein.

## DISCUSSION

Using SEREX, we previously identified a novel antigen, OX-TES-1, that is recognised by circulating antibodies present in the serum of multiple patients with DLBCL. A full-length cDNA clone encoding OX-TES-1 had been cloned and this novel gene was named *PAS domain containing 1* (*PASD1*). An alternatively-spliced form encoding a longer polypeptide has been named *PAS domain containing 1* transcript variant 2 (*PASD1_v2*), with our sequence being transcript variant 1 (*PASD1_v1*).

Data in the UniGene folder Hs.160594 suggest that the *PASD1* gene maps to chromosome band Xq28. A number of immunogenic tumour antigens with the characteristic CTA expression pattern have been mapped to this region, including MAGE-A, NY-ESO-1, LAGE-1, TRAG-3, CSAGE and SAGE (Scanlan *et al*, 2002). In addition, Xq28 has been identified as a genetic region that is altered in lymphomas and may contain lymphoma-associated oncogenes (Goyns *et al*, 1993; Vineis *et al*, 1999), one example being the high level amplification of Xq28 identified in some blastic mantle cell lymphomas (Bea *et al*, 1999).

Analyses of the gene structure indicated that the longer *PASD1_v1* cDNA appeared to have retained the sequence from intron 14, whereas this is removed in *PASD1_v2*. This alternative splicing results in the immediate introduction of a translational stop codon that truncates the PASD1_v1 protein. Many cancer-associated genes are alternatively-spliced, and it has been suggested that these may be extremely useful as cancer markers since there may be striking differences in the usage of alternatively-spliced variants between normal and tumour tissue (Caballero *et al*, 2001). There are also numerous publications reporting mRNAs containing retained intronic sequences, including the retention of CD44 introns in bladder cancer (Cooper, 1995), and the recently-described upregulation of the novel proapoptotic BH3-only splice variant of *BIM* in prostate cancer cells (Liu *et al*, 2002). It should be noted that both *PASD1* transcripts have been identified in testis, indicating that both forms are expressed in this normal tissue.

Both the PASD1 polypeptides share the same N-terminal 638-aa sequence, and analysis of the shared N-terminal protein sequence indicated that PASD1_v1 and PASD1_v2 are novel PAS domain proteins. They are most closely related to the PAS family members CLOCK and NPAS2 that play an important role in regulating eukaryotic circadian rhythms (Dunlap *et al*, 1999; Dioum *et al*, 2002). Overlapping the PAS domains was a partial aryl-hydrocarbon receptor nuclear translocator domain (ARNT), aa 3–187. ARNT is the central heterodimerization partner of several transcription factors, including those containing the aryl-hydrocarbon (dioxin) receptor (AhR) and the hypoxia-inducible factor 1alpha (HIF-1alpha). A report that the AhR/ARNT heterodimer directly associates with oestrogen receptor-*α* and -*β* is interesting in light of our identification of an LXXLL motif or nuclear receptor box in the N-terminus of the PASD1 protein that might mediate similar interactions (McInerney *et al*, 1998). While no studies have directly implicated AhR/ARNT in lymphomagenesis, it has been linked to leukaemogenesis (Salomon-Nguyen *et al*, 2000; Hayashibara *et al*, 2003). Recently, the AhR pathway has also been proposed as a novel drug target to control cell proliferation (Elferink, 2003).

Additional domains detected in the PASD1 protein suggest that this protein may be a nuclear PAS domain transcription factor. The predicted leucine zipper motif provides a potential nucleic acid-binding domain and there is a predicted nuclear localisation signal. Furthermore, proline- and glutamine-rich regions are commonly found in transcriptional activation domains, while activation domains rich in basic amino acids have also been described (Triezenberg, 1995).

Our studies of the combined expression of *PASD1_v1* and *PASD1_v2* mRNAs point towards PASD1 being a novel CTA, showing somatic tissue expression restricted to normal testis while also showing widespread expression in cancer patients. However, the *PASD1* expression levels seen in the histologically-normal tissues from patients with uterus, lung and small intestine cancer may indicate that *PASD1* expression is an early event in carcinogenesis occurring before histological changes are apparent. Indeed, the expressions of many CTAs, including those that map to Xq28, have been linked to the DNA hypomethylation that occurs early during tumorigenesis. An association between p53 hypomethylation in peripheral blood lymphocytes and the development of lung cancer among male smokers has been reported (Woodson *et al*, 2001). This raises the possibility that changes in the hypomethylation of other genes might also be detectable in non-malignant tissues. We are, however, currently unable to explain why, for example, the uterine tissue from cancer patients should show *PASD1* expression, while the tumour tissue does not, although one difference could be the cellular composition of normal and malignant tissue. Further studies will concentrate on characterising the expression of the PASD1 protein as this will have more significance as to the clinical utility of this molecule; monoclonal antibodies are currently being raised to both the short and long forms of the protein.

The *PASD1* cDNA was cloned from a testis cDNA library, therefore it was necessary to confirm that the serum reactivity of DLBCL patients resulted from the expression of this gene. RT–PCR studies confirmed the expression of *PASD1_v1* in all DLBCL-derived cell lines tested, while *PASD1_v2* appeared to be expressed only in the cell lines derived from a poor prognosis non-germinal centre subtype of DLBCL. Expression of the longer PASD1_v2 protein may be a useful subtyping marker for the identification of high-risk DLBCL patients. Interestingly, Scanlan *et al* (2002) reported that the frequency of mRNA expression of CTAs such as NY-ESO-1, MAGE-A1, MAGE-A3 and SSX-2 was higher than the humoral response observed against the protein. This is consistent with our RT–PCR data, showing expression of *PASD1_v1* in all seven DLBCL cell lines, although serum reactivity was only detected in 40% of patients. Of the four patients mounting a humoral immune response to PASD1, three (no biopsy material was available for subtyping the fourth patient) were associated with high-risk groups of DLBCL.

Until very recently, the SEREX technique had not been employed to identify B-cell lymphoma antigens. However, a study using serum from lymphoma patients, including those with DLBCL, to screen a testis library has recently been published (Huang *et al*, 2002). This study identified two known CTAs, HOM-TES-14/SCP-1 and NY-ESO-1, and two novel antigens with a restricted normal expression pattern (HOM-NHL-21 and HOM-NHL-23) that were not widely expressed in lymphomas or in solid tumours (Huang *et al*, 2002). The authors suggested that any new CTAs are likely to be expressed only infrequently in malignant tumours. This group also investigated the mRNA expression of 10 known CTAs in a range of non-Hodgkin's lymphomas and found that, while their expression in T-cell lymphomas was frequent, their expression in B-cell non-Hodgkin's lymphoma patients was comparatively rare (Xie *et al*, 2003). Such studies emphasise the importance of the identification of a novel CTA, particularly one that shows a relatively high frequency antibody response in B-cell lymphoma patients.

In conclusion, we have identified a novel CTA, PASD1, that warrants further study since its expression in both haematopoietic and nonhaematopoietic malignancies raises the possibility that this antigen may have a diagnostic or therapeutic use in a variety of cancers.
